# Supplementary material for: Causal Relationship between Aging and Anorexia Nervosa: A White-Matter-Microstructure-Mediated Mendelian Randomization Analysis
Source: Biomedicines. 2024 Aug 16;12(8):1874. doi: 10.3390/biomedicines12081874 (PMC11351342; doi:10.3390/biomedicines12081874)
Supplement: Supplementary file 1 [file biomedicines-12-01874-s001.zip › Supplementary Figure/Figure S4/Singe SNP plot of MR analysis for TL to ubm-b-1543.pdf]

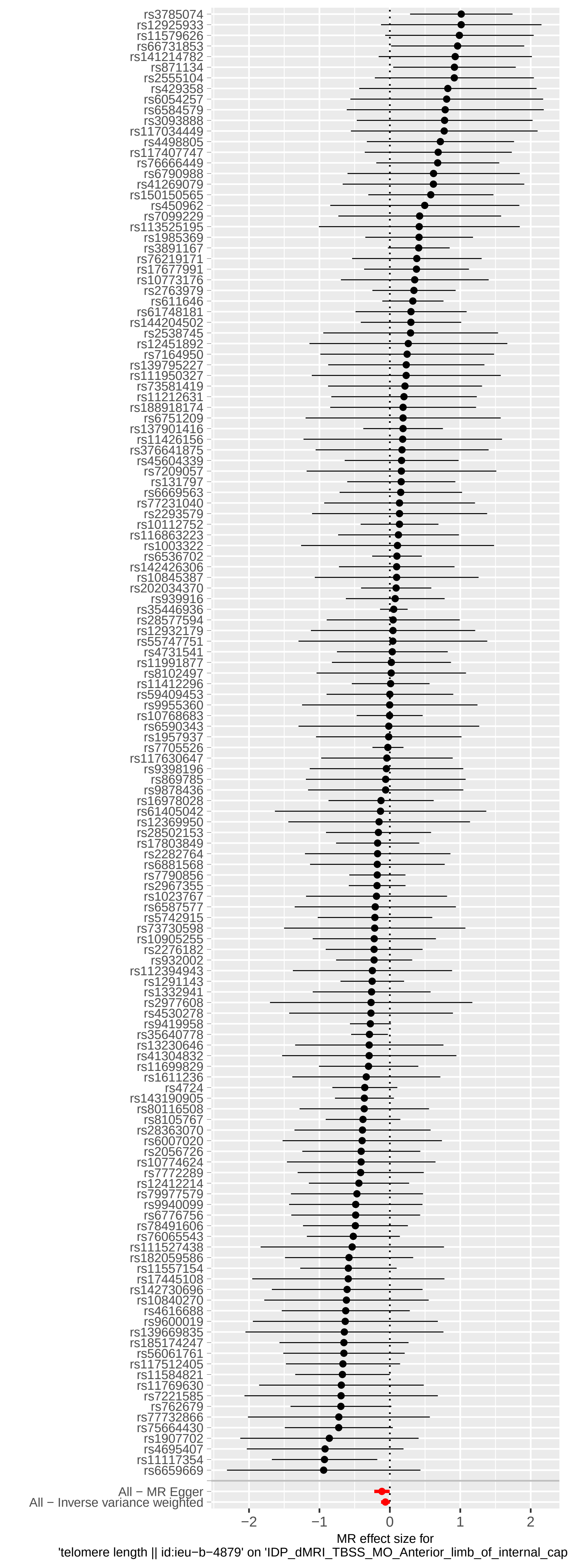
rs3785074
rs12925933
rs11579626
rs66731853
rs141214782
rs871134
rs2555104
rs429358
rs6054257
rs6584579
rs3093888
rs117034449
rs4498805
rs117407747
rs76666449
rs6790988
rs41269079
rs150150565
rs450962
rs7099229
rs113525195
rs1985369
rs3891167
rs76219171
rs17677991
rs10773176
rs2763979
rs611646
rs61748181
rs144204502
rs2538745
rs12451892
rs7164950
rs139795227
rs111950327
rs73581419
rs11212631
rs188918174
rs6751209
rs137901416
rs11426156
rs376641875
rs45604339
rs7209057
rs131797
rs6669563
rs77231040
rs2293579
rs10112752
rs116863223
rs1003322
rs6536702
rs142426306
rs10845387
rs202034370
rs939916
rs35446936
rs28577594
rs12932179
rs55747751
rs4731541
rs11991877
rs8102497
rs11412296
rs59409453
rs9955360
rs10768683
rs6590343
rs1957937
rs7705526
rs117630647
rs9398196
rs869785
rs9878436
rs16978028
rs61405042
rs12369950
rs28502153
rs17803849
rs2282764
rs6881568
rs7790856
rs2967355
rs1023767
rs6587577
rs5742915
rs73730598
rs10905255
rs2276182
rs932002
rs112394943
rs1291143
rs1332941
rs2977608
rs4530278
rs9419958
rs35640778
rs13230646
rs41304832
rs11699829
rs1611236
rs4724
rs143190905
rs80116508
rs8105767
rs28363070
rs6007020
rs2056726
rs10774624
rs7772289
rs12412214
rs79977579
rs9940099
rs6776756
rs78491606
rs76065543
rs111527438
rs182059586
rs11557154
rs17445108
rs142730696
rs10840270
rs4616688
rs9600019
rs139669835
rs185174247
rs56061761
rs117512405
rs11584821
rs11769630
rs7221585
rs762679
rs77732866
rs75664430
rs1907702
rs4695407
rs11117354
rs6659669
All − MR Egger
All − Inverse variance weighted
−2
−1
0
1
2
MR effect size for
'telomere length || id:ieu−b−4879' on 'IDP_dMRI_TBSS_MO_Anterior_limb_of_internal_cap
